# Dynamic Structural Health Monitoring of Slender Structures Using Optical Sensors

**DOI:** 10.3390/s120506629

**Published:** 2012-05-18

**Authors:** Paulo Antunes, Rui Travanca, Hugo Rodrigues, José Melo, José Jara, Humberto Varum, Paulo André

**Affiliations:** 1 Instituto de Telecomunicações and Departamento de Física da Universidade de Aveiro, 3810‐193 Aveiro, Portugal; E-Mail: pantunes@ua.pt; 2 Departamento de Engenharia Civil da Universidade de Aveiro, 3810‐193 Aveiro, Portugal; E-Mails: rui.travanca@ua.pt (R.T.); hrodrigues@ua.pt (H.R.); josemelo@ua.pt (J.M.); hvarum@ua.pt (H.V.); 3 Facultad de Ingeniería Civil de la Universidad Michoacana San Nicolás de Hidalgo, 58040 Morelia, Michoacán, Mexico; E-Mail: jmjara@umich.mx

**Keywords:** fiber Bragg gratings, fiber-based sensors, accelerometer, structural health monitoring

## Abstract

In this paper we summarize the research activities at the Instituto de Telecomunicações—Pólo de Aveiro and University of Aveiro, in the field of fiber Bragg grating based sensors and their applications in dynamic measurements for Structural Health Monitoring of slender structures such as towers. In this work we describe the implementation of an optical biaxial accelerometer based on fiber Bragg gratings inscribed on optical fibers. The proof-of-concept was done with the dynamic monitoring of a reinforced concrete structure and a slender metallic telecommunication tower. Those structures were found to be suitable to demonstrate the feasibility of FBG accelerometers to obtain the structures' natural frequencies, which are the key parameters in Structural Health Monitoring and in the calibration of numerical models used to simulate the structure behavior.

## 1. Introduction

The loss of life and the enormous damages occurred in civil engineering structure disasters during the last decades have exposed the importance of preventative actions to mitigate the effects of structural failures. Therefore, the evaluation of potential damage to structures in different permanent and/or accidental load scenarios is an important issue to be considered in rehabilitation decisions and emergency measure planning. One of the most promising techniques used nowadays for monitoring the dynamic movement of structures is the ambient vibration analysis. The analytical models are idealizations aimed at representing the response of structures subjected to vertical and/or horizontal loads, and are used to evaluate the potential risk associated to a structure. During the model creation process, several assumptions must be adopted related to uncertainty parameters, such as the real materials' strength, the elements' stiffness and ageing effects, among others. Attempts to improve the quality of analytical models often involve the use of both destructive and nondestructive material testing programs.

Ambient vibration tests have special relevance in the characterization of large civil engineering structures, such as highway bridges, where it is difficult to provide the significant level of input force required to carry out a realistic forced vibration test. Ambient vibration tests can be used for control of the different phases of a construction process and/or to acquire knowledge about the constructed structure. Structural monitoring and the application of techniques for identification of the dynamic characteristics of structures, in the time or frequency domain, allow the detection of eventual damages by the use of methodologies for assessing the structural health. Any change in the dynamic properties of a structure identified by a monitoring process could be an indication of a decrease of its load-carrying capacity.

Different monitoring processes and strategies have been extensively used for identifying the dynamic properties of buildings and bridges in several countries [1–6]. An advantage of using ambient vibration tests compared to the forced tests is the light equipment used in the former case. In these types of tests the structure vibration monitored is mainly the result of wind, micro tremors and, in the case of bridges, the circulation of vehicles or trains. The ambient vibration records can also be used to calibrate the properties of laboratory test models or to improve analytical models of buildings, bridges or other complex structures [7].

Another important area of monitoring applications in the field of Civil Engineering is in the assessment of a structure's health state. Monitoring of changes in the system frequencies is one of the more frequently employed approaches to evaluate the eventual progressive deterioration of civil infrastructures. It usually requires the use of system identification techniques [1,8,9] for continuously monitoring the health state over a short period, as discussed in [10–14]. In this way, maintenance and rehabilitation actions to prevent excessive damage of the structure can be planned, minimizing the maintenance costs for the overall lifetime of the structure, with adequate safety levels.

Structural health monitoring may require the instrumentation of several points of the structure. A larger number of monitored points may allow a more rigorous calibration of the numerical model of the structure, and a more precise definition of the eventual location of damages. This is one of the aspects to be considered in the selection of the data acquisition system and equipment. In bridges, for example, the span lengths of the structures may demand the use of sensors with good record transmission quality over long distances.

In important structures, structural health monitoring system has been also used to assess the integrity of the system [15]. In these cases, the monitoring system could be designed to trigger alarms when it detects an eventual failure mode or when a specific limit state is reached.

As stated previously, different monitoring processes have been also adopted for determining changes in the dynamic properties induced by small amplitude movements (e.g., by ambient vibration) and by large amplitude demands [16,17]. The results can be related to different damage states of a structural system.

To employ traditional electronic accelerometer technology the user must consider several limitations, such as the deployment cost, difficulty to multiplex a number of different bulky sensors, the high electromagnetic field sensitivity and the limitations associated to applications in hostile environments. To overcome these restrictions the use of optical technology is often required in the construction of accelerometers, which represents a step forward to the development of innovative monitoring schemes and systems in a large number of applications.

Fiber Bragg Gratings (FBG) have intrinsic characteristics appropriate for their incorporation in sensors, like the capability to multiplex a large number of optical sensors including accelerometers, inclinometers, pressure, relative displacement and temperature sensors into the same optical fiber. Besides that, even each individual FBG sensor presents advantages in relation to the traditional semiconductor, piezoelectric, capacitive or resistive sensors, nowadays widely commercialized. The main advantages of optical technology are the immunity to electromagnetic fields, the possibility of transmission over long distances without the need for additional amplifiers and the possibility of a significant reduction in the signal to noise ratio when compared with electrical/electronic devices [18–21]. Research on this type of sensors has been growing due to the recent demand for sensors that ensure higher reliability and robustness in high hazard environments, such as nuclear plants and oil rigs.

During the last decade we have witnessed a significant effort in the development of optical sensor-related technologies for use in structural monitoring of complex structures, such as tall buildings, bridges or dams [22–27]. Actually, a large number of research groups are working in this field and several optical accelerometers have been proposed [18,22,25,28–33], although just a few bi-dimensional accelerometers have been reported. Generally, acceleration in orthogonal directions is measured using two single axis sensors, with one positioned in each direction. This method of measuring acceleration simultaneously in the two orthogonal directions is preferred by the majority of equipment manufacturers.

The major drawback of optical sensor technology resides in the significant cost of the interrogation systems. However, to make this type of sensors cost effective several techniques have been investigated, which should allow for the cost reduction of these monitoring schemes in a near future [28,34–46].

A research group of the Instituto de Telecomunicações and of the University of Aveiro has recently proposed a biaxial optical sensor which provides, in a single module, a solution to measure, with the needed robustness and sensitivity, acceleration in two orthogonal directions. This innovative solution may be used in structural heath monitoring of large and complex structures.

This paper is organized as follows: after an introduction showing the need and advantages of SHM methods and devices, the developed accelerometer is introduced in Section 2. The monitored structures and corresponding experimental setups are exposed in Section 3. Section 4 deals with the experimental results obtained with the biaxial optical accelerometer in the dynamic monitoring of the selected structures. Finally, in Section 5, the main conclusions are presented.

## 2. Fiber Bragg Grating Based Accelerometer

A fiber Bragg grating is a passive optical device based in the refraction index modulation along the optical fiber core. This modulation can be imposed in a photosensitive optical fiber exposing it to an intense pattern of UV radiation, created by interferometric processes [22]. The modulation, in the optical fiber core, behaves as a set of reflection planes along the fiber longitudinal axis [47]. If such device is illuminated by a broadband spectral optical source, it will reflect a spectral narrow band of the incident signal, being the remaining transmitted. The reflected spectral band is centered at the Bragg wavelength, *λ_B_*, given by the first order Bragg condition [47]:

(1) $$\lambda_{B}=2n_{\text{eff}}\Lambda$$

where *n_eff_* is the optical fiber effective refractive index and Λ is the modulation period. Temperature and strain dependence for *λ_B_* results from the variation of *n*_eff_ and Λ with the temperature and mechanical deformation. Exposing the fiber with the FBG to a temperature change or/and stretching/compressing it will spectrally shift *λ_B_* proportionally to the external action. The Bragg wavelength dependency on the temperature and strain could be described as follows [22]:

(2) $$\Delta \lambda_{B}=2\left(\Lambda \frac{\partial n_{\text{eff}}}{\partial l}+n\frac{\partial \Lambda }{\partial l}\right)\Delta l+2\left(\Lambda \frac{\partial n_{\text{eff}}}{\partial T}+n_{\text{eff}}\frac{\partial \Lambda }{\partial T}\right)\Delta T$$

In Equation (2), the right-hand side first term represents the strain effect on the Bragg wavelength, and the second term represents the Bragg dependency with the temperature.

**Figure 1.**
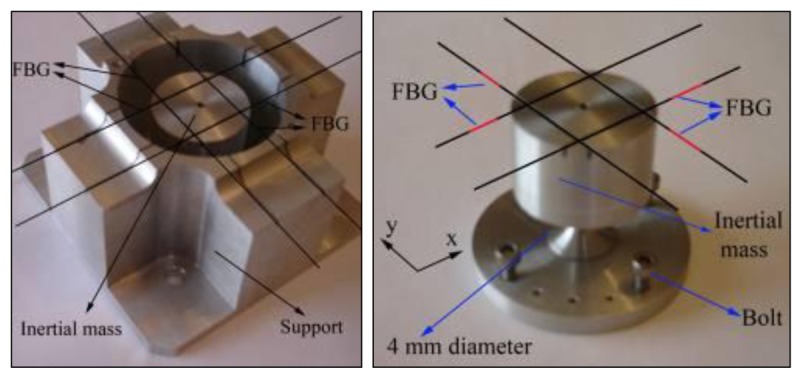
Design of the implemented accelerometer (**left**) and schematic of the central aluminum piece of the implemented accelerometer (**right**).

The implemented optical fiber accelerometer is based on four FBGs placed in opposite positions of an inertial mass. The system consists of two machined aluminum blocks which are bolted at the bottom and by the four optical fibers with Bragg gratings inscribed at the top, as illustrated in Figure 1. The simulation and experimental characterization results of the proposed sensor are presented on reference [48].

The base of the central aluminum piece is cylindrical with a diameter of 4 mm, as represented in Figure 1. In the presence of an external acceleration, the inertial mass of the sensor, presenting a diameter of 50 mm at its top, moves freely laterally. The inertial mass is free to move in two independent directions (x and y). This movement stretches or a compresses the optical fibers, resulting in a measurable shift of the FBGs Bragg wavelength, which can be related to the imposed external acceleration at the accelerometer base. With external acceleration, along the main axis direction (x or y), results in the elongation of one FBG and the compression of the one located at the opposite position. The external acceleration is obtained from the difference between the Bragg wavelengths shifts of the two gratings placed in each direction. This method increases the sensitivity to measure the acceleration by a factor of 2.

Since all four FGBs are recorded in identical optical fibers, the temperature drift will result in a similar wavelength shift in both FBGs for each direction. As the external acceleration is obtained by the difference between the Bragg wavelength shifts of the signals reflected by the two gratings, the proposed accelerometer is insensitive to environmental temperature changes.

Considering acceleration in a perpendicular direction to the FBG direction, both fibers will suffer a similar elongation, and the difference between the reflected signals wavelength shifts for both FBGs will be null. As a result, the designed accelerometer is also insensitive to the effects of cross-axis acceleration. This sensor can be applied as a low cost tool to measure acceleration in two orthogonal directions, and can be successfully used for the dynamic monitoring of several kinds of structures.

**Figure 2.**
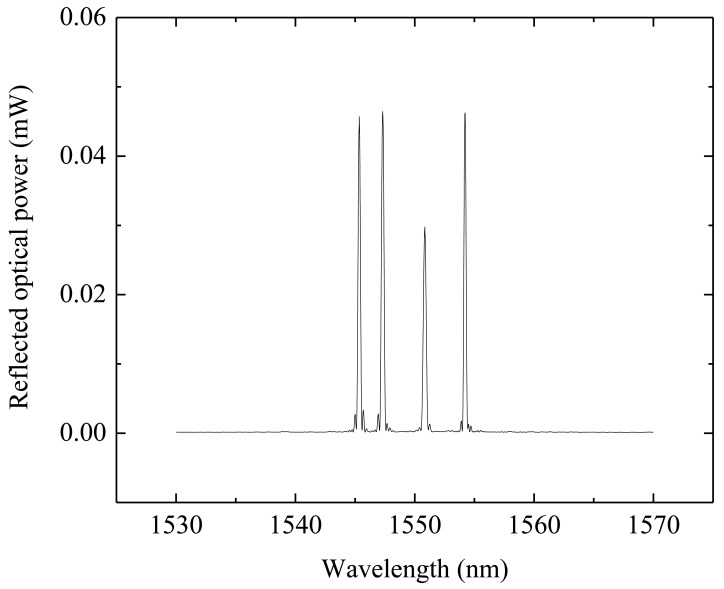
Reflection spectrum of the accelerometer output, being observed the reflection spectra for the four FBGs.

**Figure 3.**
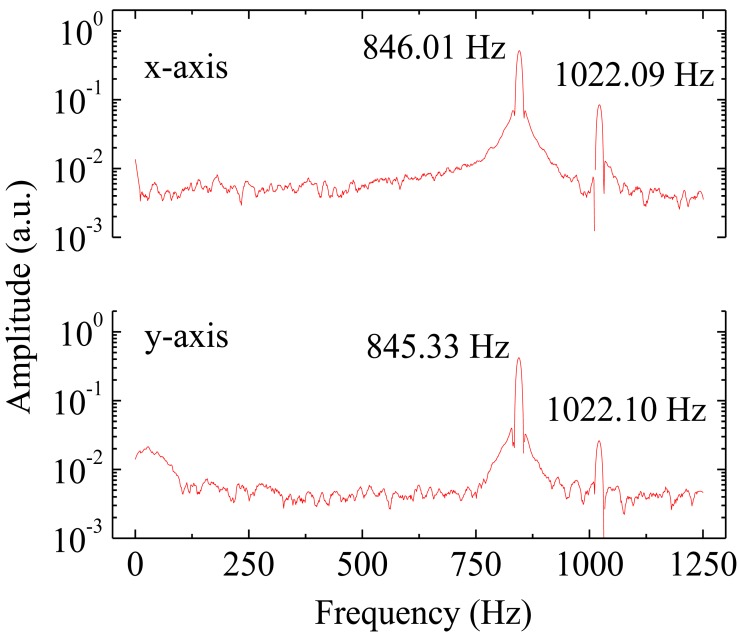
Frequency response of the accelerometer for both sensitive directions.

The four FBGs were multiplexed in the same optical fiber and the reflection spectrum measured at the sensor output is shown in Figure 2. The reflection spectrum was measured with an optical commercial interrogation unit from Micron Optics, model sm125. The experimental characterization of the sensor showed two natural frequencies in the 0–1,250 Hz range. The first resonant frequency stands for the vibration mode in the excited sensitive direction and the second one to a torsional mode. The frequency response of the sensor is presented in Figure 3, in which is possible to scrutinize the first and second resonant frequencies for each sensitive direction.

For calibration, the developed sensor was exposed to random accelerations in both sensitive directions, and its signals were compared with the signal recorded by an electronic triaxial calibrated accelerometer from Summit (model 34201A). The signals were measured with the proposed optical accelerometer and with the electronic calibrated accelerometer, for the two sensitive directions. The optical signal was acquired using an interrogation system including an optical source (model-BT1300 from Fiberamp Photonetics), an optical circulator and an optical spectrometer from Ibsen (E-Mon model I), acquiring at a rate of 900 Hz. With this approach it was possible to compare the optical sensor performance with the electrical calibrated sensor, exposing both to the same external acceleration and therefore providing a simple solution to obtain the sensitivity of the optical sensor.

The signal measured with the optical sensor is very similar to the one measured with calibrated electronic sensor, indicating an adequate operation of the optical sensor. For the x-axis and y-axis directions, sensitivities of 87.8 pm·g^−1^ and 92.4 pm·g^−1^ were obtained, respectively.

The main innovation presented by this sensor is the possibility of measuring the acceleration in two orthogonal directions simultaneously, using only one module. As a rule, a fiber optical biaxial accelerometer consists of two uniaxial optical accelerometers mounted in orthogonal directions. This method may result in a more complex, larger and more expensive system. The developed sensor offers the needed sensitivity, robustness and dimensions required for incorporation into a SHM system. The sensitivity, close to 100 pm·g^−1^ for each direction, is similar or higher than that of the current commercial uniaxial optical FBG accelerometers [33,34].

For physical protection and to facilitate its use, the accelerometer was mounted inside a steel box, with dimensions of 15.0 × 15.0 × 8.5 cm. The sensor dimensions, even with the metallic protection box, are sufficiently small to be incorporated into a structure without being intrusive or limitative to the structure functionality, considering their current proportions. On the other hand, it's still possible to miniaturize the sensor maintaining its performance, but always considering the physical limits for its construction, due to the machinery problems. However, if the proportionality on the components' dimensions is not guaranteed, the sensor response can be affected.

## 3. Monitored Structures and Experimental Setup

**Figure 4.**
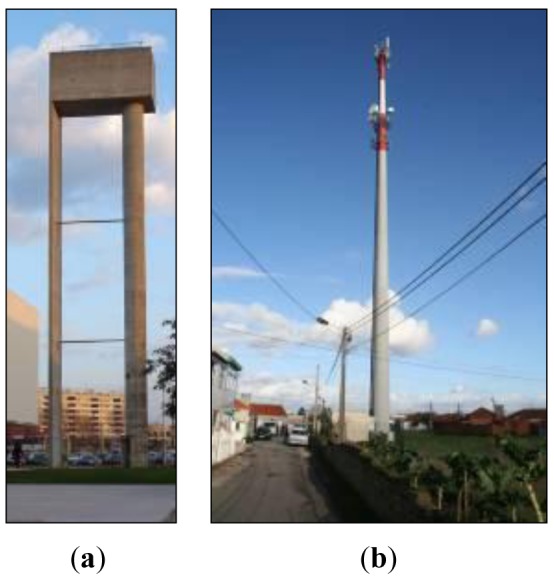
Photographs of the University of Aveiro water reservoir (**a**) and telecommunication tower (**b**).

In this section we describe the plans adopted for the dynamic monitoring of an elevated reinforced concrete water reservoir located at the University of Aveiro Campus, Portugal (shown in Figure 4(a)), and a 50 m high metallic monopole used for mobile telecommunication proposes (shown in Figure 4(b)), both located in the Aveiro region, Portugal.

The water reservoir was built between 1988 and 1989 and it is a reinforced concrete structure formed of a laminar element (with a height of 30.9 m, 4 m wide and 0.3 m thick) and a vertical element with hollow circular section (also with a height of 30.9 m, an inner diameter of 1.2 m and 0.6 m thick). The reservoir is placed above those vertical structural elements and is made of reinforced concrete with a geometry corresponding to a hollow parallelepiped which connects the two main vertical elements. The total height of the structure is 35.3 m.

Like a majority of existing structures, SHM of radio communication slender towers is crucial to assess their structural integrity and assure their design lifetime. Acceleration is an important parameter to be monitored in this type of structures. The natural frequencies of a structure can be obtained from acceleration measurements and the shifts on the natural frequency value are commonly related with the structure degradation and can be a good parameter for the evaluation of the structure physical condition. This procedure allows a preventive action, if required, cutting the costs and reducing the human injuries or loss of life. Electronic accelerometers can be used for the monitoring, nevertheless, the high electromagnetic radiation level at the top of these structures and in the neighborhood of the antennas, can easily deceive the sensor by providing wrong results and can shadow the radio operation, resulting in economic losses for the telecommunication operator. All-optical instrumentation, like that based on fiber Bragg gratings proposed in this paper, can be used as an effective solution to monitor this type of structures.

Tower typology varies across countries, related mainly to their utilization purpose and environmental actions to be considered during their design lifetime. A 50 m high monopole was chosen in this work to show the applicability of the developed sensor. In recent years, towers have became probably one of the most significant physical supports for the installation of radio apparatus, allowing the emission of electromagnetic waves and a variety of services such as television, radio and mobile communications [49,50]. Regrettably, there a number of anomalies have been observed and most of them are related to design errors, which result in hazardous structures with the possibility of collapse [51–55]. Towers are lightweight structures, with high slenderness and great flexibility, therefore dynamic analysis may be very helpful to determine the resonance response, particularly to be used on the calibration of numerical models. Those models can predict the structure response to extreme environmental catastrophes, allowing reducing maintenance costs and assuring structural safety monitoring.

The tower is rigidly fixed to a reinforced concrete semi-deep base block with base dimensions of 3.30 × 3.30 m and 3.60 m deep. The monopole is constituted by seven different modules with hexadecagonal variable cross-section.

**Figure 5.**
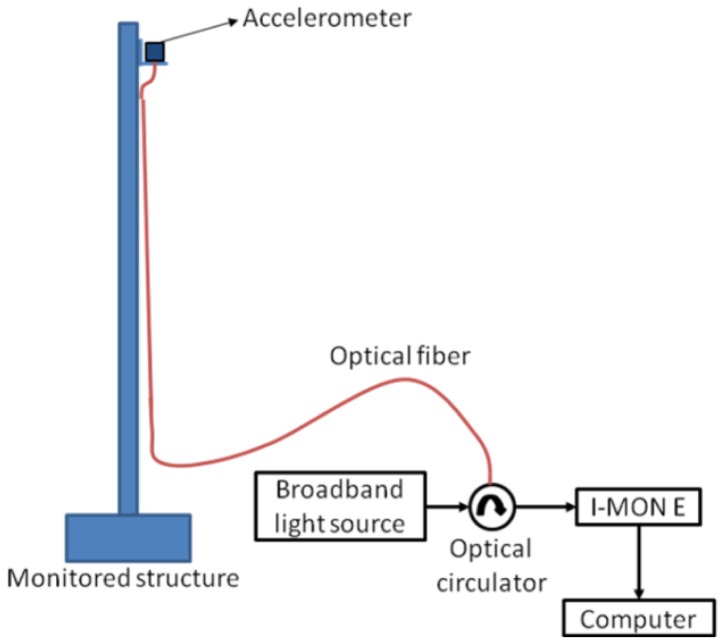
Scheme of the experimental setup used in dynamic monitoring tests.

To obtain the natural frequencies for the reservoir and for the monopole, a similar experimental setup was adopted, including the optical Bragg gratings biaxial accelerometer. The monitoring equipment includes an optical source with high spectral width, model ALS-CL-17-B-FA from Amonics, a circulator and an optical spectrometer from Ibsen, model I MON E, with an acquisition rate of 950 Hz. In the case of the reservoir, as a reference monitoring, a seismograph (GSR18 model from GeoSig) was used for data validation. Figure 5 shows the scheme of the experimental setup used in the two high slender structures.

Since the aim of the paper is to demonstrate the applicability of FBG optical sensors on the dynamic monitoring of high slender structures, a SHM plan was not fully established to undergo the evaluation of the structural state. Such a plan often involves a finite element model to support the decisions about the most appropriated location for the measurements, which could be static or dynamic. For the most successful dynamic characterization of structures, the location for the sensors installation must be calculated. This could be done estimating the structural point for which larger movements of the structure are expected, amplifying the measured signal and improving the signal-to-noise relation, or determining the points that better represent the dynamic response of the structure. In a structural evaluation plan, regular measurements are recommended to control the construction stability and/or to confirm the decay evolution. In this work, it is reported the natural frequencies measurement for the selected structures. As it is generally recognized that the structural decay of many structures could often be detected by the experimental estimation of their natural frequencies reduction, which is associated with material or structural degradation.

The reservoir top has an edge with 15 cm high, that was chosen for the application of slight horizontal mechanical impulses with a hammer (at the transverse direction), in a position at least 1.5 m away from sensors. For the telecommunication tower, the FBG biaxial accelerometer was rigidly fixed at the top of the tower, close to the antennas, and mechanical impulses were applied at the half-height of the structure and the acceleration was measured in two perpendicular directions, parallel to the ground.

## 4. Results

### 4.1. Water Reservoir

**Figure 6.**
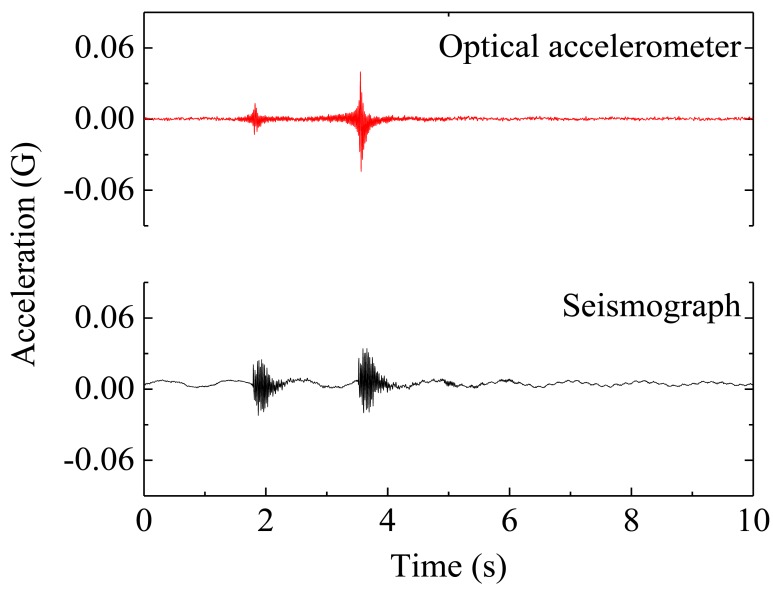
Acceleration data recorded with the optical sensor and seismograph during the application of mechanical impulses, measured in the transversal direction.

**Figure 7.**
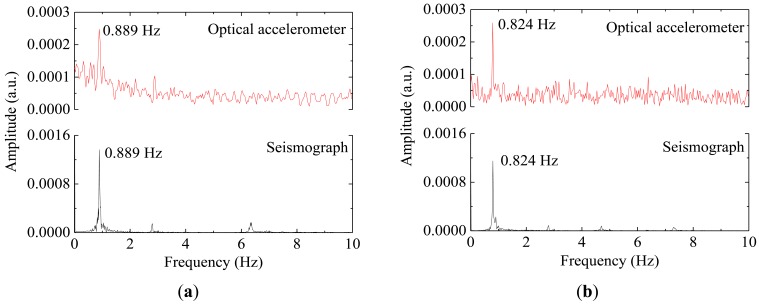
Frequency acceleration spectra from the data recorded in the transversal direction (**a**) and in the longitudinal direction (**b**).

The acceleration data, measured with two sensors (optical accelerometer and the reference seismograph), throughout a time window of 10 s is presented in Figure 6. The frequency spectra presented in Figure 7(a,b) are obtained by Fast Fourier Transform of the time-acceleration data, from which is possible to assess the natural frequencies of the structure by peak-picking.

The optical sensor clearly detects the applied impulses and the discrepancies between the measured signals with the two different sensors might be justified by the different support bearing conditions of the two sensors. While the seismograph, in spite of its weight, is merely supported on the structure, the optical accelerometer is firmly attached to the structure; these different fixing conditions can eventually allow differential movements between the structure and the seismograph. The relative difference between the values of the natural frequencies obtained with the optical sensor and the seismograph are lower than 0.06%, demonstrating the feasibility of dynamic monitoring with optical sensors for SHM of tall structures, as the concrete water reservoir studied.

### 4.2. Radio Communications Tower

**Figure 8.**
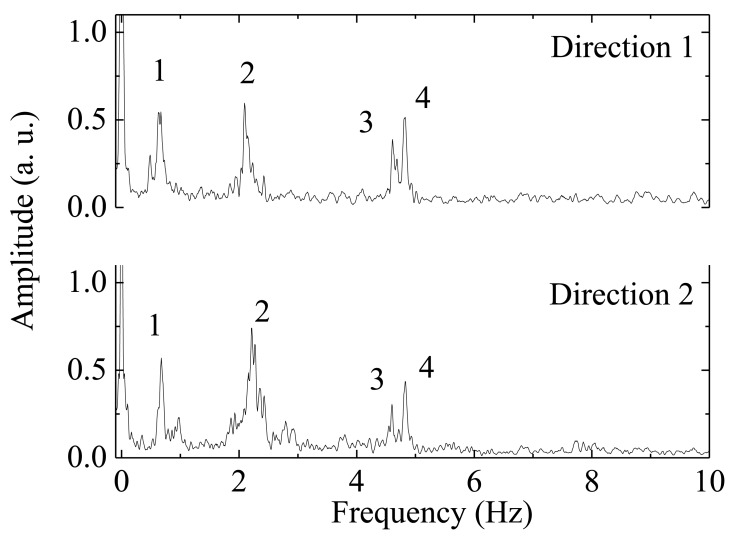
Radio communications tower frequencies spectra.

Applying a Fast Fourier Transform to the time-acceleration data recorded during the application of mechanical impulses on the tower, the eigenfrequencies of the structure were obtained from the frequency spectra by peak-picking. The frequency spectra are presented in Figure 8.

Table 1 presents the peak natural frequencies from Figure 8, obtained experimentally with the optical accelerometer in the tower, for both measured directions.

The measured values obtained with the FBG biaxial accelerometer are consistent with the results of numerical models and they are in the range of values expected for this type of structures [49,56].

### 4.3. Dynamic Monitoring During Natural Loading

**Figure 9.**
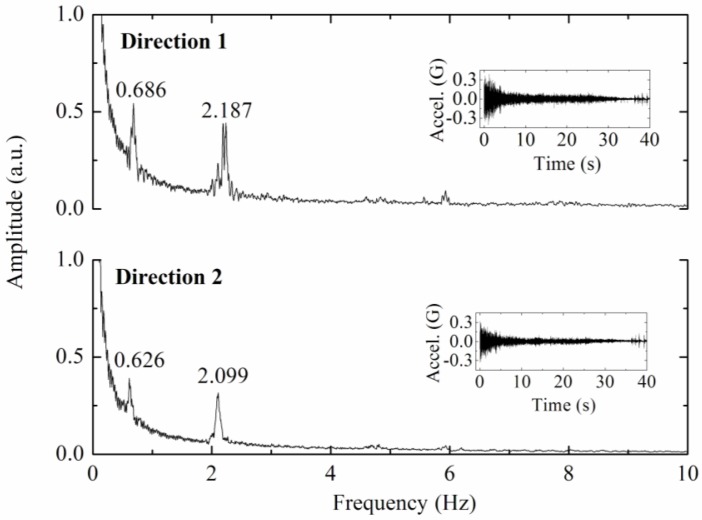
Frequency spectra obtained from the acceleration data recorded in the tower, upon a train transit. The origin corresponds to the instant when the train last wagon passed in the position perpendicular to the tower. Time-acceleration data are shown in the inset.

For certain structures, the determination of natural frequencies from the measurement of vibrations induced by ambient vibration allows useful information for dynamic characterization [57]. Given the location of the tower, close to a railway line, it was possible also to estimate the natural frequencies, from the acceleration data recorded during the passage of a train. The railway is located approximately 50 m from the tower and vibrations induced by the train transit are clearly felt at the tower. Figure 9 shows the frequency spectra obtained by Fast Fourier Transform of the acceleration data, in the inset is displayed the acceleration recorded at the top of tower during the train transit.

**Figure 10.**
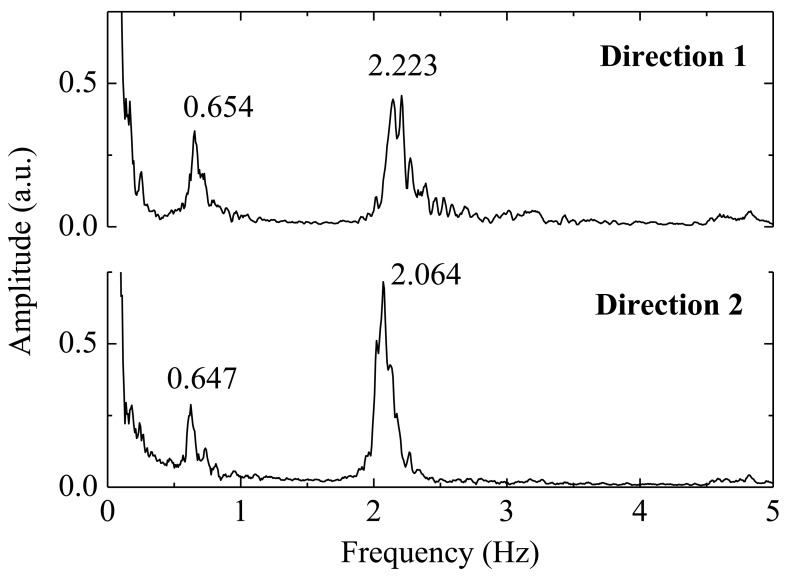
Frequency spectra obtained from the acceleration data recorded in the tower, when subjected to the wind action.

The natural frequencies obtained in the case of moving load are consistent with the ones obtained with the external load excitation. Thus, it is confirmed that it is possible to obtain the natural frequencies of such towers remotely with the proposed sensor. For the dynamic characterization, the simplest situation in terms of excitation is the registration of accelerations in the tower when subjected to purely ambient actions, such as the wind. Figure 10 shows the frequency spectra obtained by Fourier transform of the acceleration data recorded the tower, when subjected to the wind action.

Data analysis of the Figures 9 and 10 shows that the implemented sensor can be used for remote monitoring of the towers subject to ambient vibrations or to forced excitations.

## 5. Conclusions

In the work presented in this paper we have demonstrated the feasibility of dynamic SHM of tall slender structures with optical technology, namely optical FBG accelerometers. We have shown the implementation of an FBG based biaxial accelerometer and its application in the dynamic properties structures assessing. The analyzed structures are located in the Aveiro region of Portugal.

The dynamic properties of an elevated water reservoir in the longitudinal and transverse directions were determined with the results of a dynamic monitoring of the structure. Simultaneously to the measurements taken with the optical sensor on the reservoir, a seismograph was also used to identify the natural frequencies of the structure, giving a maximum relative error of 0.06%.

The results for the dynamic monitoring of the telecommunication tower showed the feasibility of using optical technology on the dynamic monitoring of tall metallic slender structures. Recurring to this technology it was possible to obtain the natural frequencies for two orthogonal directions, which could be used to predict the response of this type of structures along his lifetime.

The dynamic monitoring results showed that the presented FBG biaxial accelerometer is an excellent tool for SHM, particularly for the calibration of numerical models, not only of existing structures, but also for the optimization of new solutions. For SHM schemes, the dynamic characterization using accelerometers is commonly used, based on the natural frequencies estimation from acceleration measurements, being less expensive and intrusive than the traditional loading tests. The FBG transducers are also advantageous relative to traditional electronic sensors for long duration SHM schemes, due to the lower visual impact of using its multiplexing capabilities, which drastically decreases the cabling volume. All those advantages are relevant for every single type of structure, but for historical buildings or monuments they became of extreme importance.

## Figures and Tables

**Table 1.** Experimental natural frequencies of the tower.

**Peak number**	**Direction 1 (Hz)**	**Direction 2 (Hz)**
1	0.647	0.676
2	2.093	2.209
3	4.607	4.599
4	4.817	4.825
